# A balanced set of standardized affective environmental images for climate change research

**DOI:** 10.3389/fpsyg.2026.1944740

**Published:** 2026-10-13

**Authors:** Erkin Asutay

**Affiliations:** JEDI-Lab, Department of Behavioral Sciences and Learning, Linköping University, Linköping, Sweden

**Keywords:** affective reactions, climate change, climate visuals, environmental images, individual differences, stimulus set, affective climate images

## Abstract

Evocative visual imagery is central to research on affective pathways of environmental engagement, yet existing stimulus sets often overrepresent negative imagery and make it difficult to compare positive and negative affective responses under comparable conditions. We developed a balanced database of affective environmental images designed to support controlled comparisons of positive and negative environmental imagery. In total, 587 participants rated 203 images depicting consequences of unmitigated climate change and context-matched positive alternatives on pleasantness and activation. Participants also reported belief in human caused climate change, perceived risk of climate change, and environmental values. The image set covered a broad range of pleasantness while positive and negative images showed comparable activation levels. Across stimuli, pleasantness and activation were quadratically related, consistent with affective stimulus sets spanning over positive and negative valence. Trial-level analyses showed that stronger biospheric values and higher risk perception predicted more negative and more activating responses to negative images. For positive images, stronger biospheric values predicted more pleasant and more activating responses, whereas higher risk perception predicted lower activation. This balanced, context-matched image database provides a standardized resource for studying how affective responses to environmental imagery may shape risk perception and environmental engagement.

## Introduction

As the consequences of unmitigated climate change become increasingly evident—manifesting through extreme weather events, biodiversity loss, and resource scarcity—there is an urgent need for behavioral and lifestyle changes across global populations. This urgency underscores the importance of understanding the psychological mechanisms behind pro-environmental behavior as well as attitudes and perceptions related to climate change and sustainability issues. Even though researchers point to affective mechanisms as a significant source of motivation for engagement with environmental issues (Asutay et al., 2024; Brosch and Steg, 2021; Schneider and van der Linden, 2023), affect-driven mechanisms of pro-environmental decision-making and updating beliefs and knowledge about sustainability issues remain understudied. Critically, evocative visual images are central for investigating these affective mechanisms, testing behavioral interventions, and effectively communicating climate information. Here, we present a study of affective environmental visual images that (1) elicit a balanced range of positive and negative affective reactions, (2) contain positive and negative images matching in environmental context as well as elicited activation levels and (3) can be used by researchers, interventionists, and communicators for studying affective pathways of environmental engagement.

Affective visual imagery can significantly influence how people feel, interpret, and engage with climate change (O'Neill, 2019). Across studies, images inducing negative affect with high activation are the most commonly used in investigations and the most strongly associated with climate change as they usually depict destroyed ecosystems, extreme weather events, and destruction caused by disasters (Lehman et al., 2019; O'Neill and Smith, 2013). Negative affect can increase perceived risk of climate change (Feldman and Hart, 2018; Karlsson et al., 2023; Wang et al., 2018); while positive imagery (successful futures, mitigation efforts, and solutions) may increase perceptions of efficacy, but it may not enhance perceived threat and urgency (Chapman et al., 2016; Feldman and Hart, 2018; Hart and Feldman, 2016). The downstream consequences of these affective reactions on behavior are not well-understood. While negative emotions can motivate action, they may also lead to feelings of disengagement and overwhelm individuals. On the other hand, positive affect (including feeling hopeful and inspired) may also motivate action through an enhanced perception of efficacy, but it can also lower sense of urgency reducing engagement (Feldman and Hart, 2018; Wang et al., 2018). Understanding these affective pathways is crucial for the development of effective communication strategies and interventions to increase environmental engagement. Visual imagery plays a pivotal role in eliciting and testing these various affective mechanisms influencing engagement with sustainability issues. Consequently, the development of stimulus sets that are standardized for their affective qualities is useful for researchers and communicators studying the influence of visual imagery on affective responses and behavioral outcomes.

Currently existing curated sets—complete with pleasantness, activation, and relevance ratings—usually lack a balanced representation of positive and negative images, containing more negative than positive stimuli (Lehman et al., 2019; Ottavi et al., 2021). Negative images typically depict impacts of unmitigated climate change (disasters, ecosystem destruction, and pollution), while positive images depict nature, solar panels and wind turbines, climate protests, and politicians and public figures. This disparity in both pleasantness and content may limit researchers’ ability to compare the effects of positive and negative affective reactions to environmental images on behavioral outcomes. Critically, it may be difficult to source stimuli matching activation levels across different levels of pleasantness, complicating the interpretation of findings. To fill this gap, I sourced a balanced array of images eliciting positive and negative affective reactions across similar activation levels. Moreover, the difference in content for positive and negative images may be another confounding factor. To match the range of content between negative and positive stimuli, first negative images were curated based on various consequences of unmitigated climate change for humans and other ecosystems. Then, positive images were selected matching the context of negative images (for details, see Methods; Table 1). This balanced approach may help researchers and interventionists studying and comparing affective pathways of belief update and behavior change.

**Table 1:** Description of stimuli.

Negative	Positive	Description	N
Destroyed forests and woodland areas (incl. Biodiversity loss and wildfires)	Healthy and thriving forest ecosystems	Images depict forests and woodland areas, no humans. Negative images depict destroyed and damaged forest ecosystems (18 images) and wildfires (15 images). Positive images include healthy forests including animals (18 images) and, to balance wildfire images, 15 landscape shots of healthy forests and woodland areas are included.	66 (32%)
Destroyed ocean ecosystems	Healthy and thriving ocean ecosystems	Every image is taken underwater and does not contain any human aspect. Positive images show corals and other animals while negative images are barren.	22 (11%)
Bad air quality in urban areas	Clear and good air in cities	Every image includes man-made structures and/or urban context. Negative images show smog and no vegetation, while positive images depict clear air and trees.	24 (12%)
Extreme weather events (incl. Human suffering and destruction)	Non-extreme weather events (incl. People enjoying weather)	Every image includes a human aspect (i.e., humans, man-made structures and/or urban context). Negative images show extreme weather events (heatwave, flood, hurricane, destruction of property), while positive images show non-extreme weather events (sunny, rainy or snowy weather, no destruction, people enjoying the weather)	38 (19%)
Food and water scarcity	Food and water abundance	Every image includes an aspect about food scarcity (e.g., devastated crops, drought, people receiving food aid) or food abundance (e.g., healthy farmlands, people harvesting crops, fresh vegetables and fruit)	28 (14%)
Destruction of indigenous communities (incl. Forced migration)	Thriving indigenous and rural communities (incl., pristine nature)	Images depict human aspects and/or nature consequences in non-westernized contexts. Positive images include indigenous people and pristine nature, while negative images show natural degradation, destroyed rural communities, migrating people, and refugee camps.	26 (13%)

Equal number of positive and negative images were included for each group.

In the current study, 587 individuals rated their affective reactions on pleasantness and activation dimensions in response to a diverse set of 203 environmental images. Participants’ belief in man-made climate change, perceived risk of climate change, and environmental values were assessed. The data was analyzed to ensure that images induced a wide range of pleasantness, positive and negative stimuli covering similar levels of activation, which is critical for a balanced set of affective environmental images. Further, affective reactions to each image were analyzed to study individual differences in responding based on perceived risk of climate change and biospheric values.

## Method

### Stimulus selection

Images were selected from open-access crowd-sourced stock image platforms (pexels.com and unsplash.com) and previously published affective climate image databases (Lehman et al., 2019; Ottavi et al., 2021). During image selection, we focused on 6 consequences of unmitigated climate change: (1) destruction of forests and woodland areas (including wildfires and biodiversity loss), (2) destruction of ocean ecosystems, (3) worsening air quality in urban areas, (4) more frequent extreme whether events, (5) food and water scarcity, (6) destruction of rural and indigenous communities (including destruction of nature and forced migration). Within each of these 6 domains, negative (e.g., bleached coral reef on a barren ocean floor) and positive (e.g., healthy and thriving coral reef ecosystem) imagery were curated (Table 1; for representative images see Supplementary materials). The stimulus set contained 204 images. However, due to an error in randomization during the survey, one image was not shown to any participant. Thus, the final image set consisted of 203 images, of which 22 were from Lehman et al. (2019) and 13 were from Ottavi et al. (2021).

### Participants

603 participants were recruited in Prolific. All participants listed English as their primary language and had 100% approval rate on the platform. 3 participants were excluded due to a failed attention check and 13 were removed because their responses were flagged by the bot detection algorithm on the survey platform (Qualtrics.com). The final sample included 587 participants (273 male, 305 female, 7 non-binary, 2 chose not to disclose) between 18 to 78 years of age (M = 38, SD = 12.9). Each participant viewed and rated 30 randomly selected images (15 positive and 15 negative) in a random order. Each image was rated by 78–93 individuals (median = 87). Participants gave informed consent at the beginning of the survey. Median completion time was 11.4 min (Mean = 12.8 min; SD = 5.6 min).

### Materials and procedure

After reporting age and gender identification, participants rated 30 randomly selected images on pleasantness, activation, and relevance dimensions (see Supplementary materials). Pleasantness and activation scales were presented with the question: “How does this image make you feel?” Pleasantness scale ranged from 1 (Unpleasant) to 9 (Pleasant) with mid-point (5) marked as ‘neutral.’ Activation scale ranged from 1 (sleepy) to 9 (alert). After rating how the image made them feel, participants rated perceived relevance of the image to climate change (“How relevant do you think this image is to climate change?”) on a 5-point scale ranged from 1 (not at all) to 5 (very much). We included the relevance scale to provide a comparable stimulus set to previously published datasets (Lehman et al., 2019; Ottavi et al., 2021).

Following image ratings, we assessed participants’ belief in human caused climate change, perceived risk of climate change, and values relevant to environmental attitudes (E-SVS; Steg et al., 2014). Participants first responded to the following question: “Based on what you know, which of the following statements best describes your view on climate change? Choose one of the options.” The options were: (1) I believe the climate is not changing; (2) I am not sure whether the climate is changing or not; (3) I believe that the climate is changing, but that it has little to do with human conduct; (4) I believe that the climate is changing, and that it is largely the result of human conduct.

Participants then rated how much risk they thought climate change poses to themself (*To what extent do you perceive climate change presents a risk to you?*), to others (*To what extent do you perceive climate change presents a risk to other people?*), and to the non-human populations of the earth (*To what extent do you perceive climate change presents a risk to non-human populations of the world (animals, insects, etc.)?*) on visual analogue scales ranging from 0 (no risk at all) to 100 (extreme risk). An aggregate measure of perceived risk of climate change was calculated from the three items (*ω_t_* = 0.89). Finally, we assessed values relevant to environmental attitudes (E-SVS; Steg et al., 2014). The survey includes 16 values, wherein participants rate the importance of each value as a guiding principle in their lives on a scale from −1 (against my principles) to 7 (highest importance). The scale also contained verbal labels at 0 (not important), 3 (important), and 6 (very important). The 16 items were aggregated into four types of values: hedonic (*ω_t_* = 0.8), egoistic (*ω_t_* = 0.82), altruistic (*ω_t_* = 0.86), and biospheric (*ω_t_* = 0.93).

### Data analysis

Analyses were done in R 4.5.2. Stimuli, data, and the analysis script are available at OSF.^1^ I computed descriptive statistics and Pearson bivariate correlations between individual level values and risk perception, as well as between mean image ratings (i.e., pleasantness, activation, and relevance). Next, the relationship between pleasantness and activation ratings were investigated using bivariate correlations and polynomial regressions. Furthermore, I analyzed the influence of biospheric values and perceived risk of climate change on affective reactions to positive and negative environmental images using linear mixed models. The models contained image valence (positive vs. negative), biospheric values, and perceived risk as main effects. To study differences in responding, I included 2 interaction terms: *image valence X biospheric values* and *image valence X perceived risk*. The model also contained trial number to control for linear trends in responding during the survey. Further, to account for between-participants and between-stimuli variations, random intercepts over individuals and images were also included. All reported *p*-values were adjusted using Benjamini-Hochberg false discovery rate unless stated otherwise.

## Results

### Descriptive statistics of the sample

82.8% (486 individuals) agreed that humans’ conduct is the main reason of climate change, while 11.1% (65 individuals) did not think the humans are the main reason of climate change. Finally, 1.4% (8 individuals) did not think the climate was changing and 4.7% (28 individuals) were unsure. Perceived risk of climate change was positively associated with having biospheric (r(587) = 0.45, *p* < 0.001) and altruistic (r(587) = 0.39, *p* < 0.001) values. Having egoistic and hedonic values did not significantly correlate with perceived risk of climate change (for means, standard deviations, and correlations, see Table 2).

**Table 2:** Means, standard deviations, and correlations with confidence intervals (*N* = 587).

Variable	Mean (SD)	Risk perception	Biospheric	Altruistic	Egoistic
Risk perception (0–100)	68.3 (23.6)				
Biospheric (−1–7)	5.23 (1.54)	0.45 ** [0.35, 0.53]			
Altruistic (−1–7)	5.48 (1.36)	0.39 ** [0.29, 0.49]	0.66 ** [0.59, 0.72]		
Egoistic (−1–7)	2.55 (1.48)	−0.06 [−0.18, 0.05]	0.06 [−0.05, 0.18]	0.05 [−0.06, 0.17]	
Hedonic (−1–7)	4.02 (1.5)	0.03 [−0.08, 0.15]	0.14 ** [0.02, 0.25]	0.11 * [0.00, 0.23]	0.49 ** [0.4, 0.58]

SD is standard deviation. Pearson correlation coefficients are presented. Values in square brackets indicate the 95% confidence intervals. The confidence intervals and p-values are corrected for multiple comparisons using false discovery rate. *p < 0.05, **p < 0.001.

### Image ratings

For each image, average pleasantness, activation, and relevance ratings were computed. The average ratings induced a wide range of pleasantness (1.36 to 8.35; M = 5.00, SD = 2.32), while activation ratings were more concentrated around the mid-point of the scale (M = 5.06, SD = 0.58, min = 3.77, max = 6.78). Relevance ratings were distributed over almost the entire scale range (M = 3.2, SD = 0.78, min = 1.67, max = 4.68).

A moderate negative association was found between pleasantness and activation (r(203) = −0.44, *p* < 0.001). To further investigate the relationship between pleasantness and activation ratings over the entire stimulus set, we ran polynomial regressions, predicting activation based on pleasantness. Equation 1 shows linear and quadratic models.

$$\begin{array}{l} \;\text{Linear model}:\text{Activation}\sim 1+\text{Pleasantness}\\ \text{Quadratic model}:\text{Activation}\sim 1+\text{Pleasantness}+\text{Pleasantnes}s^{2} \end{array}$$ (1)

The analyses showed that the second order model provided a significantly better fit compared to the linear model according to a significant likelihood ratio test (chisq(1) = 94.3, *p* < 0.001). The mean pleasantness and activation ratings together with the best fitting relationship is shown in Figure 1A. The quadratic relationship between pleasantness and activation is typical in affective stimulus sets that cover a wide range of pleasantness (e.g., Bradley and Lang, 2007; Kurdi et al., 2017; Lang et al., 1997; Yang et al., 2018).

**Figure 1:**
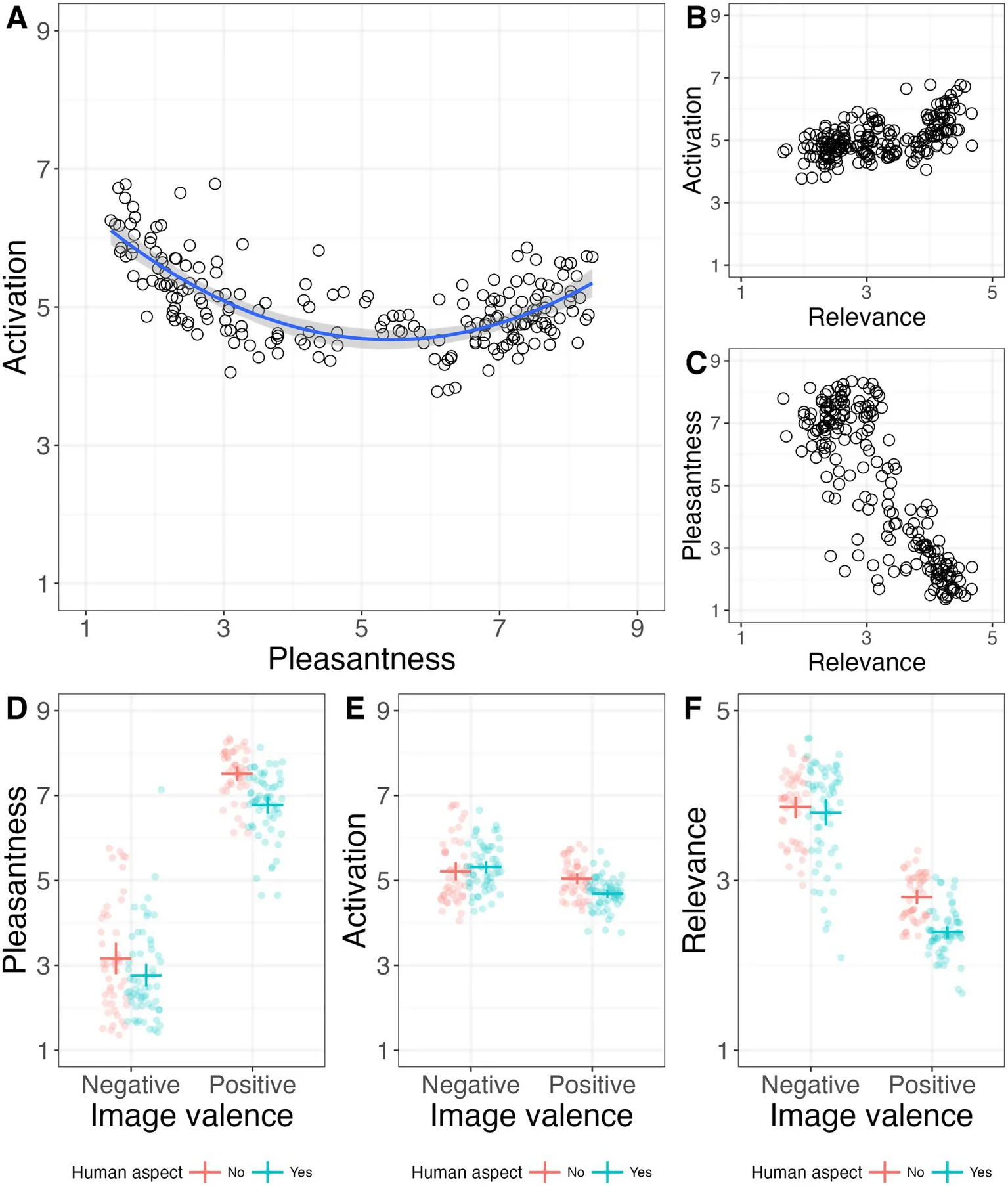
Image rating analyses. **(A)** Distribution of images over the 2-dimensional pleasantness-activation space. The blue line shows the second order relationship between activation and valence ratings. Shaded area represents 95% confidence interval. **(B,C)** Scatterplots showing how relevance ratings change with pleasantness and activation. **(D–F)** Differences in affective reactions and relevance ratings based on image valence and whether the image contained any human aspect.

Moreover, relevance ratings were correlated with both pleasantness (r(203) = −0.84, *p* < 0.001) and activation (r(203) = 0.55, *p* < 0.001). These correlations are in line with previous results showing individuals’ tendency to rate negative environmental images (e.g., nature destruction, wildfires, and extreme weather events) as more relevant to climate change (Figures 1B,C).

### The role of human content

In addition, I studied differences in image ratings based on image valence (positive vs. negative) and whether the image contained a human aspect. 88 images (43%) did not contain any human aspect (i.e., humans, man-made structures, and/or urban contexts). These stimuli contained images of ocean ecosystems and forest and woodland areas. Pleasantness, activation, and relevance ratings were submitted to two-way ANOVAs (image valence X human aspect; see Figures 1D–F). All *p*-values were Bonferroni corrected. Both image valence (*F*(1,199) = 1,157, *p* < 0.001, generalized-eta2 = 0.82) and human aspect (*F*(1,199) = 21.1, *p* < 0.001, generalized-eta2 = 0.1) had significant effects on pleasantness ratings, indicating that positive images and images with no human aspect induced more pleasantness in comparison to negative images and those containing a human aspect. Interaction effect was not significant but separate tests for positive and negative images indicated that human aspect difference was significant only for positive images (*p* < 0.001). Image valence had a significant effect on activation ratings (*F*(1,199) = 32, *p* < 0.001, generalized-eta2 = 0.14) indicating that positive images on average induced lower activation. Human aspect effect was not significant (*p* > 0.1). The interaction effect was significant (*F*(1,199) = 8.7, *p* = 0.004, generalized-eta2 = 0.04). The separate tests indicated that human aspect had a significant effect only for positive images (*p* < 0.001), indicating that for positive images containing a human aspect induced lower activation compared to positive nature images. Both image valence (*F*(1,199) = 440, *p* < 0.001, generalized-eta2 = 0.69) and human aspect (*F*(1,199) = 15.2, *p* < 0.001, generalized-eta2 = 0.07) had significant effects on relevance ratings, indicating that positive images and images containing a human aspect were rated as less relevant than negative images and those containing no human aspect. The interaction effect was also significant (*F*(1,199) = 8.2, *p* = 0.005, generalized-eta2 = 0.04). Further tests indicated that human aspect effect was significant only for positive images (*p* < 0.001).

Taken together, no significant differences were found between negative stimuli depicting human vs. nature consequences. However, for positive stimuli, those depicting nature (i.e., no human aspect) were rated as more pleasant, more activating, and more relevant to climate change.

### The influence of biospheric values and perceived risk of climate change on affective reactions to environmental images

Finally, I analyzed individual differences in affective reactions to environmental images based on participants’ biospheric values and perceived risk of climate change using linear mixed models predicting pleasantness and activation ratings at the trial level. The models had the following fixed effects: image valence (positive vs. negative), biospheric values, perceived risk, image valence X biospheric values interaction, image valence X perceived risk interaction, and trial number. The model also included random intercepts over individuals and over images. On average, pleasantness ratings were significantly higher for positive images (B = 2.22, 95%CI = [1.86, 2.58], *p* < 0.001), while activation ratings did not significantly differ between positive and negative images (B = −0.03, 95%CI = [−0.35, 0.29], *p* > 0.5). Main effects of biospheric values and perceived risks as well as their interactions with image valence were significant for both pleasantness and activation ratings (all at *p* < 0.001 level; see Table 3 for coefficient estimates). To ease interpretation, the main effects and interactions were visualized (Figure 2). The results show that with increasing biospheric values individuals reported greater negative affect and higher activation for negative images and greater positive affect and higher activation for positive images. Higher risk perception was associated with greater negative affect and higher activation in response to negative images, and lower activation for positive images. Hence, affective reactions to negative environmental images were similarly related to biospheric values and risk perception. However, biospheric values and risk perception had different influences on affective reactions to positive environmental images, which induced stronger positive affect (with higher activation) as biospheric values increased and lower activation as risk perception increased.

**Table 3:** Coefficient estimates and confidence intervals for linear mixed models predicting pleasantness and activation responses.

Predictors	Pleasantness		Activation	
	Estimate	95% CI	Estimate	95% CI
(Intercept)	3.90 **	3.57–4.23	3.92 **	3.46–4.38
Image valence [positive]	2.22 **	1.86–2.58	−0.03	−0.35 – 0.29
Biospheric values	−0.07 **	−0.11 – −0.03	0.11 **	0.05–0.17
Perceived risk	−0.06 **	−0.09 – −0.03	0.09 **	0.04–0.14
Image valence [pos] × Biospheric values	0.21 **	0.18–0.24	0.09 **	0.05–0.13
Image valence [pos] × Perceived risk	0.07 **	0.04–0.09	−0.16 **	−0.20 – −0.13
Trial number	−0.01 **	−0.01 – −0.00	−0.00	-0.00 – 0.00
N participants	586 _pid_		586 _pid_	
N images	203 _IM_		203 _IM_	
N total	17,610		17,610	
Marginal R^2^/conditional R^2^	0.56/0.72		0.03/0.26	

CI is confidence interval. Non-standardized estimates are presented. *p*-values are corrected for multiple comparisons using false discovery rate. *p < 0.05, **p < 0.001.

**Figure 2:**
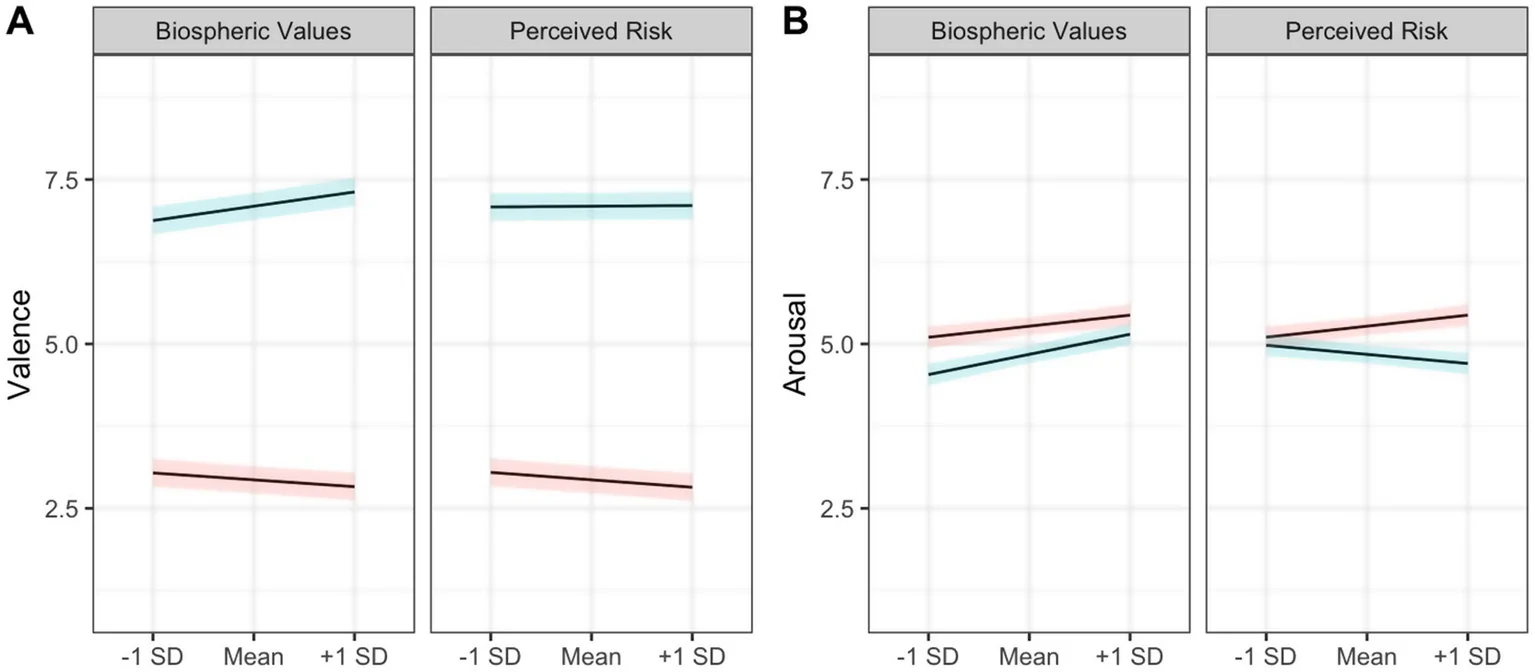
The effect of biospheric values and risk perception on affective reactions to positive (blue) and negative (red) environmental images. Shaded areas represent 95% confidence intervals.

## Discussion

The current study is on affective reactions to environmental visual stimuli inducing both positive and negative affective reactions providing a balanced approach. In comparison to previous stimulus sets, the pleasantness in the current set is centered around the neutral (mid-point) with a larger distribution evidenced by a greater standard deviation (current set: M = 5.00, SD = 2.32; Lehman et al., 2019: M = 4.79, SD = 1.38; Ottavi et al., 2021: M = 4.03, SD = 1.51). An additional feature of the stimuli is that pleasantness and activation are quadratically related enabling future researchers to source stimuli with matching activation levels for pleasant and unpleasant images. Finally, positive and negative images were also matched by content. Since our aim was to balance positive and negative stimuli to be used in climate communications focusing on consequences of whether humans would meet the urgency of the climate crisis, we started with several consequences of unmitigated climate change to be depicted in negative stimuli with varying degrees of severity and then matched the context in positive stimuli (for details, see Table 1). Taken together, this balanced approach of sourcing activation- and context-matched positive and negative stimuli may be helpful in studies on affective pathways for enhancing environmental engagement.

In addition, we show that people with higher risk perception and stronger biospheric values also had stronger affective reactions to negative stimuli (i.e., more unpleasant and higher activation). This is very much in line with previous research showing that higher perceived climate risk and stronger biospheric values tend to predict stronger negative climate affect (Karlsson et al., 2023; Lehman et al., 2019). However, the evidence on how positive affective reactions to environmental images differ is scant in comparison. Here, we showed that biospheric values and climate risk perception predict different affective reaction patterns for positive environmental images. Stronger biospheric values predicted stronger positive affective reactions (greater pleasantness and higher activation), while higher perceived climate risk predicted lower activation induced by positive images. Biospheric values are closely associated with the subjective sense of one’s relationship with nature (Christens et al., 2025; Martin and Czellar, 2017), which has cognitive, affective, and experiential dimensions of how people relate to natural environments (Lumber et al., 2017). Heigheted connection to nature is related to more positive environmental attitudes, greater likelihood of engaging in pro-environmental behavior, increased positive affect and well-being (Bratman et al., 2012; Zeng et al., 2025). This heightened feeling of nature connectedness may be one explanation for the differences in affective reactions to positive images. Our study is the first to show this differing affective reaction patterns to positive environmental images, further underscoring the importance to adopt a balanced approach in studying affective reactions elicited by environmental stimuli.

The current study aimed at validating affective qualities of a set of environmental images. However, several limitations should be noted. First, while the stimuli induced a wide range of pleasant and unpleasant affective reactions, the relevance ratings were not balanced. In line with previous results, participants found negative images being more relevant to climate change compared to positive images. This is not surprising when considering that climate change is inherently a negative issue signaling a looming threat image. Therefore, it is challenging to create stimulus materials that induce both positive emotions and are relevant to climate change. However, pleasant images in the current dataset may be combined with vignettes situating the image in a successful mitigation scenario to enhance their perceived relevance. Second, although normed affective ratings of stimuli showed a reasonable spread, there is individual variation in how people respond to images. Apart from the association between affective reactions and biospheric values or risk perception, individual history with climate events exacerbated by climate change (e.g., wildfires, flash floods, hurricanes, heat waves) could moderate affective reactions. The current study did not control for this factor. Future studies should consider asking individuals to self-report previous experiences and/or collecting geographical location data to control for regions experiencing frequent negative climate events. Finally, the current study only considered still images, and not other media formats. While perceptually richer media formats like video or virtual reality can offer more immersive experience and have been shown to reduce psychological distance and increase risk perceptions compared to static pictures (Meijers et al., 2023; Santoso et al., 2025), still images remain practical and a widely used tool offering greater experimental control. Standardized stimulus sets also allow for matching stimuli in affective dimensions that is more difficult to achieve with interactive media, making them well-suited for isolating specific affective and contextual features. However, still images lack the temporal and multisensory richness that other forms of media offer.

## Conclusion

The current study offers a validated set of environmental images that overcomes a key limitation of existing stimulus sets: the disproportionate representation of negative, high-activation imagery relative to positive content. By matching positive and negative images on both activation level and environmental context, the dataset enables researchers to isolate the specific contribution of affective valence to environmental engagement. Beyond this methodological contribution, the finding that biospheric values and risk perception relate differently to affective reactions to positive versus negative stimuli suggests that interventions could benefit from tailoring the stimulus materials to audience characteristics rather than defaulting to a one-size-fits-all approach. The dataset is well-suited to study affective pathways to belief updating, message-framing, and the design of stimulus material for interventions. At the same time, several limitations should be considered in applied contexts: The stimuli necessarily lack the temporal dynamics, narrative structure, and immersive qualities increasingly utilized in real-world climate communications (Santoso et al., 2025; Schäfer, 2025). Additionally, affective ratings were collected in a controlled study context, and future research should examine affective reactions when stimuli are embedded in more ecologically valid communication contexts combined with textual or narrative framing.

## Data Availability

The datasets presented in this study can be found in online repositories. The names of the repository/repositories and accession number(s) can be found at: OSF: https://osf.io/wup5m/ or GitHub: https://github.com/erkinasutay/Affective-Environmental-Images---normed-dataset.

## Footnotes

1
https://osf.io/wup5m/
