# Supplementary material for: A balanced set of standardized affective environmental images for climate change research
Source: Front Psychol. 2026 Oct 13;17:1944740. doi: 10.3389/fpsyg.2026.1944740 (PMC13710019; doi:10.3389/fpsyg.2026.1944740)
Supplement: Supplementary file 1 [file Supplementary_file_1.DOCX]

**Supplementary Materials**

**Visuals of survey items**

**Figure S 1.** Participants assessed each picture based on pleasantness, activation, and relevance scales. (A) First participants were asked to rate how each image made them feel. (B) After they reported pleasantness and activation, they were asked to rate how relevant each image was to climate change on a separate screen. The image was presented on each screen together with the question and scale(s).

**Representative images for each category**

| **Destruction of forests including wildfires** | | **Healthy and thriving forests** | |
| --- | --- | --- | --- |
|  |  |  |  |
| **Destroyed ocean ecosystems** | | **Thriving ocean ecosystems** | |
|  |  |  |  |
| **Bad air quality in cities** | | **Good breathable air in cities** | |
|  |  |  |  |
| **Extreme weather events** | | **Non-extreme weather events** | |
|  |  |  |  |
| **Food scarcity** | | **Food abundance** | |
|  |  |  |  |
| **Destruction of indigenous communities (incl. forced migration)** | | **Thriving of indigenous communities** | |
|  |  |  |  |
